# A novel non-invasive electromagnetic extendable intercalary endoprosthesis: a proof-of-concept study

**DOI:** 10.3389/fbioe.2024.1400428

**Published:** 2024-07-18

**Authors:** Siyi Huang, Jiake Yang, Xinyu Li, Xiaodong Tang, Tao Ji

**Affiliations:** ^1^ Musculoskeletal Tumor Center, Peking University People’s Hospital, Beijing, China; ^2^ Beijing Key Laboratory of Musculoskeletal Tumor, Beijing, China; ^3^ Beijing AK Medical Co., Ltd., Beijing, China

**Keywords:** extendable endoprosthesis, intercalary endoprosthesis, electromagnetic drive, limb length discrepancy, non-invasive extendable endoprothesis

## Abstract

**Introduction:** Femur and tibia are the most commonly affected sites for primary malignant bone tumors in children. The wide resection of the tumor frequently requires the physis to be resected. The normal growth of the unaffected limb will result in a significant limb length discrepancy at skeletal maturity. To compensate for this resulting LLD, different generations of extendible endoprostheses have been developed. Non-invasive extendable prostheses eliminate the need for surgical procedures and general anesthesia, enabling gradual and painless lengthening. Currently available non-invasive extendable prostheses focus on joint reconstruction, and no case series analysis of intercalary non-invasive extendable prosthesis has been reported. Therefore, we have designed a novel non-invasive electromagnetic extendable intercalary endoprosthesis.

**Methods:** *In vitro* mechanical experiments and *in vivo* animal experiments were conducted.

**Results:** *In vitro* experiments have confirmed that the prosthetics can extend at a constant rate, increasing by 4.4 mm every 10 min. The average maximum extension force during prosthetic elongation can reach 1306N. In animal *in vivo* experiments, the extension process is smooth and non-invasive, and the sheep is in a comfortable state.

**Discussion:** The *in vitro* and *in vivo* animal studies provide evidence to support the extension reliability, laying the foundation for future large-scale validation experiments.

## Introduction

Osteosarcoma and Ewing sarcoma are the most common primary malignant bone tumor in children. The incidence accounts for approximately 10% of all malignant tumors in children. The most commonly affected sites are the femur and tibia. Limb salvage surgery has now become the main treatment for patients with bone tumors (Weitao et al., 2012). Tumors frequently involve the distal femur or proximal tibia, thus requiring the resection of the physis or growth plates. The normal growth of the unaffected limb will result in a significant limb length discrepancy (LLD) at skeletal maturity. Different generations of the extendible endoprosthesis (EPR) have been developed to compensate for the resulting LLD (Huang et al., 2023).

Invasive extendable prostheses can be extended through an open invasive surgical procedure. Modular constructs are believed to provide the benefit of enhanced structural integrity of the prosthesis during maximum lengthening. The drawback lies in the necessity for a surgical procedure with extensive exposure for each lengthening event. This may result in excessive scar tissue formation and an elevated susceptibility to infection. Minimally invasive extendable prostheses can be extended through nail-tract incisions. Nevertheless, the drawbacks include the potential for expansion mechanism failure, prosthesis failure at maximum lengthening, and postoperative nail-tract infections (Nystrom and Morcuende, 2010). The latest non-invasive extendable prosthesis, eliminates the need for surgical procedures and general anesthesia to facilitate lengthening. Stanmore JTS noninvasive prosthesis (produced by Stanmore Implants Worldwide Ltd., UK) features an internal lengthening mechanism activated by an external electromagnetic field, enabling smooth and painless lengthening can be undertaken in the outpatient clinic (Dukan et al., 2022).

With the improvement of preoperative imaging examination and surgical osteotomy accuracy, some patients who previously needed joint replacement can now undergo joint preservation surgery (Guder et al., 2017; Takeuchi et al., 2019; Li et al., 2023). Especially, with advancement of 3D printing technology which enables manufacture highly custom-made endoprosthesis and metallic porous structure which facilitating bone ingrowth, joint preservation limb salvage for bone tumor resection increased in recent years (Li et al., 2023). Available non-invasive extendable prostheses focus on joint reconstruction, and no case series analysis of intercalary non-invasive extendable prosthesis has been reported. Thus, we design a novel, non-invasive electromagnetic extendable intercalary endoprosthesis.

**FIGURE 1:**
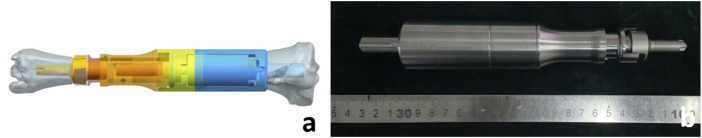
**(A)**, internal structure of the endoprosthesis; **(B)**, image of the endoprosthesis.

The endoprosthesis shaft consists of two parts that slide relatively during the lengthening procedure (Figure 1). The two stemmed ends of the endoprosthesis are secured into the remaining bone segment using cement. The main shaft includes a magnetic disc, a gearbox, and a power screw. The magnetic disc is connected to the gearbox, while the gearbox is connected to a threaded screw that links to the inner telescopic segment. When the screw rotates, it causes the inner and outer segments of the telescoping shaft to separate and extending. The magnetic disc rotates when the prosthesis is placed in a rotating electromagnetic field. The external drive unit creates the rotating electromagnetic field by a power unit, which operates from a single-phase 220-V power source. To verify the extension mechanism and mechanical stability of the prosthesis, both *in vitro* and *in vivo* animal experiments were conducted.

## Materials and methods

### *In vitro* experiment

**FIGURE 2:**
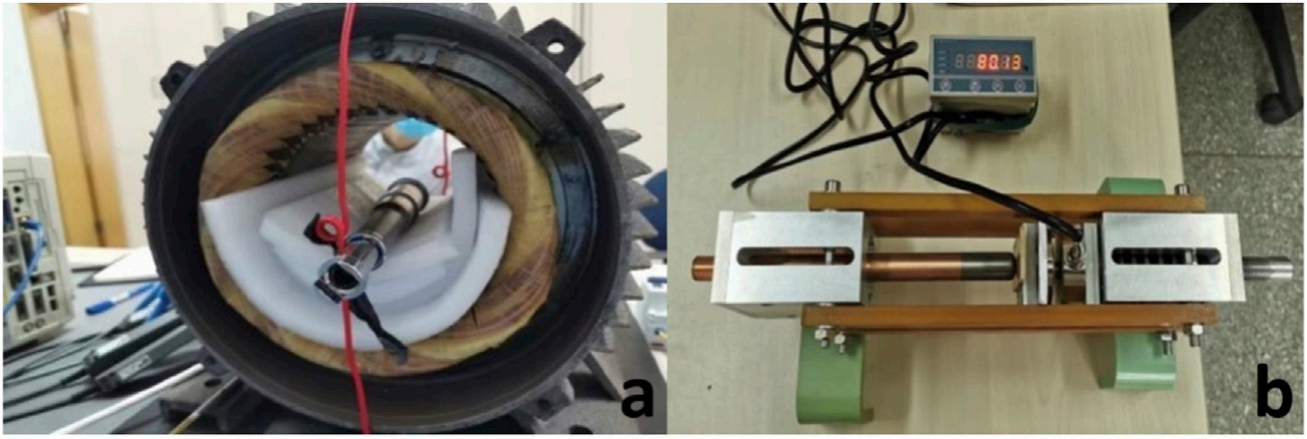
**(A)**, endoprosthesis placed in the center of the external drive unit; **(B)**, extension force testing device.

The *in vitro* mechanical experiments were conducted to investigate the extension rate and maximum extension force. The prosthesis was put at the center of the external drive unit (Figure 2A). Extension operation was started by launching the external drive unit. The magnetic generator was stopped every 10 min, and the extension length was measured. The experiment was repeated 6 times. Linear regression was used to calculate the relationship between the extension time and extension length. Then, the prosthesis was placed in the extension force testing device (Figure 2B). The maximum extension force at intervals of 5 mm for each extended length was recorded. The experiment was repeated 6 times.

### *In vivo* experiment

Sheep provide a reliable surrogate for human clinical conditions, with bone mineral composition, macro- and microstructure, remodeling capacity, and biomechanics very similar to those of humans. The load on the entire hind limbs of sheep is approximately half of the load encountered by humans during a gait cycle (Reichert et al., 2009). Therefore, we chose small-tailed Han sheep weighing about 50 Kg for the experiment.

The intercalary prosthesis was designed based on sheep CT scan data and simulated mid-shaft defects in the tibia. To induce anesthesia, a single intravenous dose of 5 mg/kg 1% Provive was injected. Then tracheal intubation was performed and a respirator machine was connected. 2% isoflurane in a mixture of oxygen/air (40:60) was used to maintain anesthesia. After general anesthesia, the sheep were placed supinely on the operation table using bandage fixation. An intercalary bone defect of 16 cm was made on the tibia and was reconstructed with the intercalary prosthesis. The stems at both ends of the prosthesis were fixed to the tibia through bone cement. Lengthening and shortening tests were performed immediately post-surgery, at 4 weeks post-surgery, and at 12 weeks post-surgery. The sheep limb was X-rayed during the assessment period to ensure the length of the prosthesis. The sheep were euthanized at 12 weeks post-surgery. The method of euthanasia for sheep was excessive intravenous injection of pentobarbital. The prosthesis was retrieved. HE staining slices were made from the bone callus around the prosthesis.

## Results

### *In vitro* experiment

**FIGURE 3:**
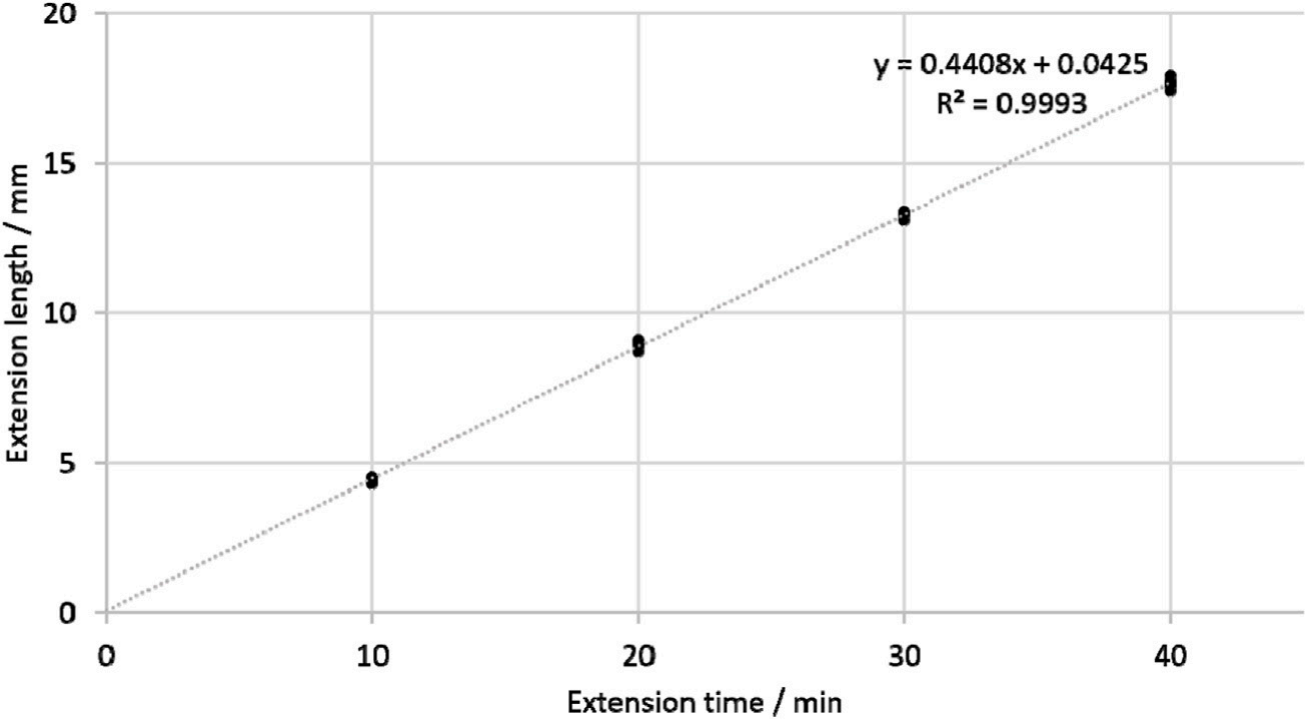
Relationship between extension time and extension length.

**FIGURE 4:**
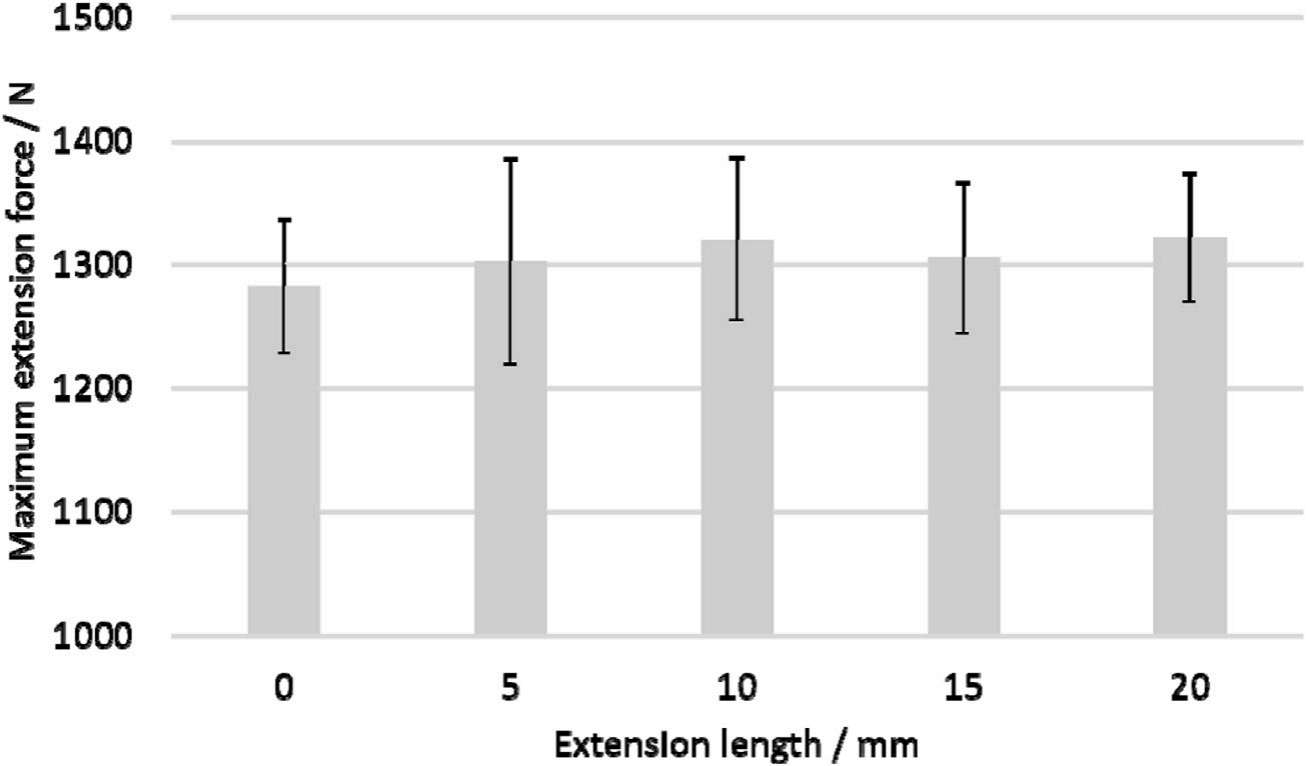
The maximum extension force under different extension length.

The fitted regression equation of extension time (T/minute) and extension length (L/mm) was as follows: L = 0.4408T + 0.0425 (Figure 3). The regression coefficient was 0.9993. Under the rotating magnetic field, the prosthesis was extended by 4.4 mm every 10 min. The high regression coefficient indicated that the extension process was very smooth. The average maximum extension force was 1,306 ± 67N (Figure 4), which is sufficient to counteract the tension of soft tissue and extend by approximately 5 mm both in sheep and human (Meswania et al., 1998).

### *In vivo* experiment

After the surgery, the prosthesis was extended a 1.8 mm-length within 4 min. Then, we shortened the prosthesis to its initial length to reduce the wound tension. The sound of the gearbox could be heard through a stethoscope placed on the surface of the sheep’s leg in order to monitor the working status of the gearbox (rotating of the magnetic disc) (Supplementary Material S1). The wound healed well, and the sheep gradually resumed walking.

**FIGURE 5:**
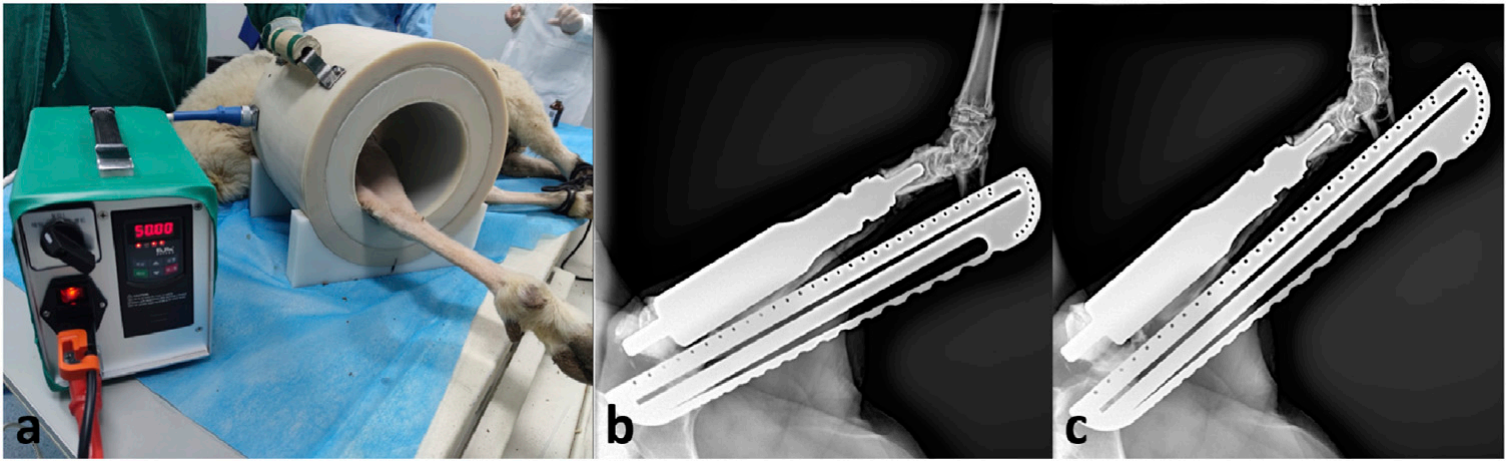
Lengthening procedure at 4 weeks after surgery. **(A)**, the sheep leg was placed in the device; **(B)**, radiograph before lengthening; **(C)**, radiograph after lengthening.

In the second lengthening procedure, the prosthesis was extended for 4.4 mm, with the lengthening process taking 10 min. Subsequently, the prosthesis was shortened for 3.2 mm. The net elongation in this experiment was 1.2 mm (Figure 5). Then the sheep was allowed to ambulate freely.

**FIGURE 6:**
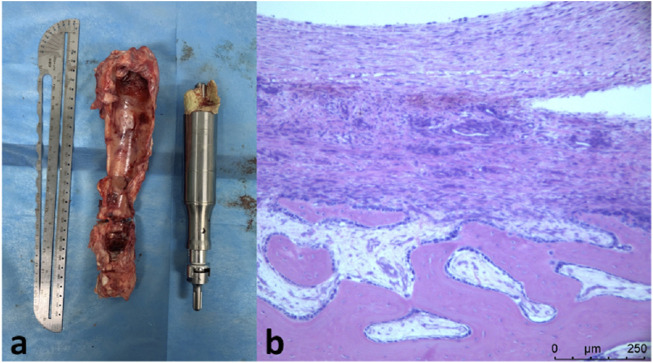
**(A)**, retrieved prosthesis and bone callus; **(B)**, H&E staining showing newly-formed bone callus with no metal particles or foreign body granulomas.

In the third lengthening process, the prosthesis was extended for 4.4 mm. The total elongation of the endoprosthesis was 5.6 mm. The prosthesis has been functioning normally during three lengthening process. The affected limb of the sheep could bear weight and walk. No complication of the prosthesis has been observed. There were no metal particles or foreign body granulomas in the HE slices (Figure 6).

## Discussion

Osteosarcoma and Ewing sarcoma are the most common primary malignant bone tumor in children. Tumors typically grow at the metaphyseal ends of long bones such as the distal femur and proximal tibia (Andreou et al., 2015; Ferguson and Turner, 2018). Limb salvage surgery has now become the main treatment for patients with bone tumors (Grinberg et al., 2020). When the joint surface is not invaded, we can excise the tumor-affected bone segment to preserve the joint. The reconstruction of bone defects mainly involves two methods. One is non-biological reconstruction, which typically involves the use of prosthetics (Smolle et al., 2019). The other method is biological reconstruction (such as allograft (Zucchini et al., 2022), vascularized (Hsu et al., 1997) or nonvascularized fibular graft (Kaewpornsawan and Eamsobhana, 2017), distraction osteogenesis (Lesensky and Prince, 2017), recycling tumor-bearing autograft (Tsuchiya et al., 2005)). Due to the inherent growth potential of the unaffected limb being higher than that of the affected limb, patients may experience a discrepancy in limb length when the bones mature.

The following method is commonly used to address postoperative limb length discrepancies in patients. The initial approach involves combining epiphysiodesis fixation with distraction osteogenesis and providing long-term support and protection using an external fixator (Watanabe et al., 2013). This method is direct and effective, but it often presents issues such as extensive trauma, prolonged recovery periods, postoperative infections, nonunion, and, most significantly, a higher risk of nerve damage, which contradicts the philosophy of limb salvage treatment. However, it cannot be denied that this procedure effectively resolves postoperative limb length discrepancies and maximally preserves the patient’s own bones (Scales, 1983). The first-generation lengthening prosthesis was designed by Dr. Scales and Stanmore in 1976. This prosthesis requires open surgery, which entails significant trauma, causing considerable discomfort, especially for patients who require multiple lengthening procedures (Lex et al., 2021). A related study indicates that the infection of the prosthesis and postoperative complications, such as loosening of the prosthesis, is relatively high (Schindler et al., 1997). The second generation of lengthening prostheses is minimally invasive. Its unique extendable screw structure allows the lengthening procedure to be completed through a small incision, but similar to the criticisms of the first-generation lengthening prostheses, it still requires the procedure to be performed in the operating room under anesthesia (Unwin and Walker, 1996). The latest generation of lengthening prostheses is designed to minimize trauma to patients even further. These prostheses contain telescopic segments that can slide past each other when placed in a specific condition, such as an electromagnetic field. By placing the prosthesis in a rotating electromagnetic field, the lengthening procedure can be performed. The lengthening is steady and painless, without the need for anesthesia or an incision (Dotan et al., 2010). Compared to the previous two generations of lengthening prostheses, the design of non-invasive extendable prostheses greatly reduces issues such as the increased risk of infection associated with surgical procedures. Additionally, it alleviates pain during the lengthening process and reduces the anxiety patients may experience when undergoing the lengthening procedure (Jeys et al., 2005; Henderson et al., 2011).

Here are several non-invasive expandable prostheses that have been applied clinically, along with the results of clinical follow-up: Wilkins RM et al. studied Phoenix expandable prosthesis. Seven Phoenix prostheses were used to reconstruct bone defects in six patients. After the implantation of the prostheses, a total of 21 extension procedures were performed in six patients. According to statistics, the average extension time was 20–30 s, with an average extension amount of 8 mm. No patients experienced acute complications during the extension procedure, but most patients still require oral analgesics during the extension process (Wilkins and Soubeiran, 2001). However, it has already been phased out by the market. Said Saghieh and colleagues reported their 7-year experience using the Repiphysis^®^ implant—all patients undergoing non-invasive lengthening procedures achieved successful elongation. But more than two-thirds of patients have developed complications, which mainly focus on infections and mechanical issues (Saghieh et al., 2010). The Juvenile Tumour System (JTS; Stanmore Implants Worldwide, Stanmore, United Kingdom) was another type of non-invasive extendible prosthesis and was first used in 2002. Hwang et al. conducted a study on 34 pediatric patients using the Stanmore prosthesis for defect reconstruction. Their early results suggest that the Stanmore prosthesis can be lengthened non-surgically. However, there still exists a relatively high infection rate (Hwang et al., 2012).

We have developed a novel non-invasive electromagnetic extendable intercalary endoprosthesis, and conducted *in vitro* and *in vivo* animal validation. There is no case series analysis of intercalary non-invasive extendable prosthesis. The application of 3 Stanmore JTS non-invasive extendable intercalary prostheses was reported (Gundavda and Agarwal, 2019). Both the novel prosthesis and JTS prosthesis utilize electromagnetic drive technology. However, the gear reduction ratio of the novel prosthesis is different from the JTS prosthesis. The magnet and gearbox of the novel prosthesis are fully sealed within a titanium alloy chamber, which has passed rigorous *in vitro* testing and meets national standards, whereas the JTS prosthesis does not have this sealed structure. The biocompatibility of the prosthesis is not the main focus of this study. Therefore, no serum metal ion test was conducted after long-term animal feeding. *In vivo* experiments on biocompatibility and ion release can be further refined in subsequent experiments.

The study confirmed the reliability and usability of the endoprosthesis. According to Meswania’s publication (Meswania et al., 1998), at 6 mm extension the load on the prosthesis due to soft tissues and muscles in human varied between 42 N and 1513 N with a mean of 476 N. There was a linear relationship between extension and load. The average maximum extension force of the novel prosthesis is 1,306 ± 67 N, which is sufficient to counteract the tension of soft tissue and extend by approximately 5 mm. The *in vitro* and *in vivo* animal studies provide evidence to support the extension reliability, laying the foundation for future large-scale validation experiments.

## Data Availability

The raw data supporting the conclusions of this article will be made available by the authors, without undue reservation.
