# Supplementary material for: A novel non-invasive electromagnetic extendable intercalary endoprosthesis: a proof-of-concept study
Source: Front Bioeng Biotechnol. 2024 Jul 18;12:1400428. doi: 10.3389/fbioe.2024.1400428 (PMC11291365; doi:10.3389/fbioe.2024.1400428)
Supplement: Supplementary file 1 [file Table1.DOCX]

Recording of the working status of the gearbox.

0-3, prepare to place the stethoscope.

3-10s, normal extension.

10-20s, gearbox deceleration.

20-27s, gearbox acceleration.

27-30s, normal extension
